# Supplementary material for: Role and mechanism of NCAPD3 in promoting malignant behaviors in gastric cancer
Source: Front Pharmacol. 2024 Apr 22;15:1341039. doi: 10.3389/fphar.2024.1341039 (PMC11070777; doi:10.3389/fphar.2024.1341039)
Supplement: Supplementary file 11 [file DataSheet2.ZIP › GSEA/Transcipt factor/my_analysis.Gsea.1599461814734/NFMUE1_Q6.html]

Details for gene set NFMUE1\_Q6[GSEA]

|  || Dataset | filtered\_dataset.sample\_info.cls#WT\_versus\_NCAPD3\_MUT |
| Phenotype | sample\_info.cls#WT\_versus\_NCAPD3\_MUT |
| Upregulated in class | WT |
| GeneSet | NFMUE1\_Q6 |
| Enrichment Score (ES) | 0.343782 |
| Normalized Enrichment Score (NES) | 1.6427062 |
| Nominal p-value | 0.019933555 |
| FDR q-value | 0.51190245 |
| FWER p-Value | 1.0 |
Table: GSEA Results Summary

  

Fig 1: Enrichment plot: NFMUE1\_Q6      
 Profile of the Running ES Score & Positions of GeneSet Members on the Rank Ordered List

  

| SYMBOL | TITLE | RANK IN GENE LIST | RANK METRIC SCORE | RUNNING ES | CORE ENRICHMENT || 1 | 51585 | PCF11 | 3 | 1.212 | 0.0701 | Yes |
| 2 | 55170 | PRMT6 | 32 | 0.981 | 0.1083 | Yes |
| 3 | 23248 | RPRD2 | 33 | 0.977 | 0.1665 | Yes |
| 4 | 3654 | IRAK1 | 69 | 0.856 | 0.1922 | Yes |
| 5 | 23338 | JADE2 | 71 | 0.855 | 0.2424 | Yes |
| 6 | 6421 | SFPQ | 87 | 0.818 | 0.2803 | Yes |
| 7 | 6925 | TCF4 | 132 | 0.768 | 0.2942 | Yes |
| 8 | 7248 | TSC1 | 158 | 0.718 | 0.3189 | Yes |
| 9 | 22845 | DOLK | 269 | 0.618 | 0.2761 | Yes |
| 10 | 4281 | MID1 | 294 | 0.595 | 0.2942 | Yes |
| 11 | 3149 | HMGB3 | 331 | 0.575 | 0.3024 | Yes |
| 12 | 55870 | ASH1L | 436 | 0.512 | 0.2577 | Yes |
| 13 | 23468 | CBX5 | 484 | 0.485 | 0.2526 | Yes |
| 14 | 140775 | SMCR8 | 487 | 0.480 | 0.2798 | Yes |
| 15 | 3184 | HNRNPD | 508 | 0.470 | 0.2933 | Yes |
| 16 | 64324 | NSD1 | 534 | 0.455 | 0.3023 | Yes |
| 17 | 55339 | WDR33 | 551 | 0.445 | 0.3173 | Yes |
| 18 | 9993 | DGCR2 | 552 | 0.445 | 0.3438 | Yes |
| 19 | 10274 | STAG1 | 633 | 0.406 | 0.3101 | No |
| 20 | 29890 | RBM15B | 651 | 0.400 | 0.3216 | No |
| 21 | 128061 | C1orf131 | 738 | 0.356 | 0.2806 | No |
| 22 | 5898 | RALA | 813 | 0.309 | 0.2454 | No |
| 23 | 8904 | CPNE1 | 814 | 0.308 | 0.2638 | No |
| 24 | 3005 | H1F0 | 880 | -0.294 | 0.2343 | No |
| 25 | 1195 | CLK1 | 920 | -0.349 | 0.2268 | No |
| 26 | 8780 | RIOK3 | 948 | -0.367 | 0.2292 | No |
| 27 | 84919 | PPP1R15B | 979 | -0.394 | 0.2309 | No |
| 28 | 1983 | EIF5 | 1219 | -0.595 | 0.0934 | No |
| 29 | 283232 | TMEM80 | 1321 | -0.740 | 0.0644 | No |
Table: GSEA details [plain text format]

  

Fig 2: NFMUE1\_Q6      
 Blue-Pink O' Gram in the Space of the Analyzed GeneSet

  

Fig 3: NFMUE1\_Q6: Random ES distribution      
 Gene set null distribution of ES for **NFMUE1\_Q6**
